# Cytomegalovirus (CMV) seroprevalence in the adult population of Germany

**DOI:** 10.1371/journal.pone.0200267

**Published:** 2018-07-25

**Authors:** Raskit Lachmann, Anna Loenenbach, Tim Waterboer, Nicole Brenner, Michael Pawlita, Angelika Michel, Michael Thamm, Christina Poethko-Müller, Ole Wichmann, Miriam Wiese-Posselt

**Affiliations:** 1 Immunization Unit, Robert Koch-Institute, Berlin, Germany; 2 Postgraduate Training for Applied Epidemiology, Robert Koch-Institute, Berlin, Germany; 3 European Programme for Intervention Epidemiology Training, ECDC, Stockholm, Sweden; 4 Charité – University Medicine Berlin, Berlin, Germany; 5 Division Molecular Diagnostics of Oncogenic infections, Infection, inflammation and Cancer Program, German Cancer Research Center (DKFZ), Heidelberg, Germany; 6 Department of Epidemiology and Health Reporting, Robert Koch-Institute, Berlin, Germany; University of St Andrews, UNITED KINGDOM

## Abstract

**Background:**

Infection with cytomegalovirus (CMV) remains asymptomatic in most immunocompetent hosts, but is the leading cause of congenital viral infection worldwide and can be life-threatening in immunocompromised individuals. We aimed to assess CMV seroprevalence in a nationally representative sample of adults in Germany and to identify sociodemographic factors associated with CMV seropositivity.

**Methods:**

Blood samples from 6552 participants (18–79 years) of the “German National Health Interview and Examination Survey 1998”, a population-based sample of the adult population in Germany, were tested for the presence of CMV antibodies using an Ig-multiplex assay. Weighted seroprevalence was calculated and weighted binomial regression was used to identify factors associated with CMV seropositivity.

**Results:**

Overall CMV seroprevalence was 56.7% (95%CI: 54.8–58.7%), with a higher seroprevalence in women (62.3%) than in men (51.0%). Seroprevalence increased with age: from 31.8% to 63.7% in men and from 44.1% to 77.6% in women when comparing the 18–29 with the 70–79 year age-group, respectively. CMV seroprevalence in women of childbearing age (18–45 years) was 51.7%. Factors significantly associated with CMV seropositivity were age, country of birth, smoking status, education, living in northern Germany and number of household members. In addition, having attended child care was associated with seropositivity in men, and number of siblings and living in East Germany in women.

**Conclusion:**

Our results indicate that half the women of childbearing age were susceptible for primary CMV infection during pregnancy. CMV screening during pregnancy and informing seronegative women about CMV risk reduction measures could prevent congenital CMV infections with its serious consequences.

## Background

Cytomegalovirus (CMV) is a human herpesvirus which is prevalent worldwide with an estimated seroprevalence of 45% to 100% in the general population [1]. After primary infection the virus remains latent. Transmission can occur through contact with CMV-infected body fluids both during primary infection or episodes of reactivation from latency. CMV infections are usually asymptomatic in immunocompetent hosts but can cause life-threatening complications in immunocompromised individuals [2]. CMV infection is a major hazard in patients with AIDS and other immune disorders, transplant recipients, individuals admitted to intensive-care units, and to some extent in elderly people. However, the highest disease burden is due to congenital CMV infection [2–4]. Worldwide, congenital CMV infection is the leading cause of neurological damage in children and is associated with growth retardation, hearing loss, permanent disabilities and microcephaly [5, 6].

Despite this considerable public health burden, few women are aware of congenital CMV infection [7–9]. Educating women about CMV transmission and preventive hygiene behaviour can significantly reduce primary CMV infections during pregnancy and thereby congenital CMV infections [10–14]. A vaccine would be necessary to significantly and permanently reduce congenital (and other) CMV infections. To date, there is no licensed vaccine available that protects against CMV. However, several vaccine candidates are currently being tested in clinical trials [15–17]. A vaccine against CMV was classified as a top priority by "The National Vaccine Advisory Committee" in the US in 2004, based on the estimation that the disease burden of congenital CMV infection is as high as the disease burden due to congenital rubella before the introduction of rubella vaccinations [18, 19]. Representative epidemiological data on the CMV susceptibility of the population are essential for decision making in the fields of public health and primary prevention through immunization. Since there has been no population-based CMV-specific Ig seroprevalence data available for German adults, the aims of this study were to estimate CMV seroprevalence in the adult population in Germany and to identify socio-demographic and lifestyle factors that are potentially associated with CMV seropositivity.

## Methods

### Study population

The German National Health Interview and Examination Survey 1998 (GNHIES98) was the first nationwide representative survey on the health status of Germany’s adult population after the German reunification in 1990. A nationwide two-stage clustered sample design with a selection of study points was used. The sampling of the 120 study points was done with a probability proportional to community size and federal state. Persons aged 18–79 years stratified by sex and age-group from the local population registers were subsequently sampled [20]. The net sample of the GNHIES98 consisted of 7124 persons (response: 61%) from 120 study points. Subjects were eligible if they were familiar with the German language and were able to complete the questionnaires.

Although there was no law or regulation on Ethic Committees in Germany at the time of the conduct of the study, the study, including the analysis of the CMV-specific Ig data, was approved by the Board of the Federal Commissioner for Data Protection Berlin, Germany. The study was conducted according to the Federal and State Commissioners for Data Protection guidelines. Informed written consent and assent were obtained from all participants and all data were fully pseudomized before analysis.

### Survey methods

In total, 7124 participants were examined at local study centers and blood samples were taken from a total of 6757 individuals (94.8%). For the study on CMV-specific Ig prevalence, blood samples of 6552 (92.0%) participants were available. Information on socio-demographic and lifestyle variables were obtained by standardized self-administered questionnaires. Place of residence was categorized into North, Middle and South Germany (Region I) as well as into former East and West Germany (Region II). Country of birth was categorized as Germany or other than Germany. Education was categorized into three levels (low, medium, high) according to the “International Standard Classification of Education”. Smoking status was categorized into never smoking, former smoking and current smoking. As a proxy for the number of children, the number of people under the age of 18 currently living in the household was used, since there were no data on gravidity or parity available. As a proxy for siblings, the number of children grown up with was used.

### Laboratory methods

Blood samples from the GNHIES98, which were stored at the Robert Koch Institute in Berlin at -70°C, were shipped to the German Cancer Research Center (DKFZ) in Heidelberg, Germany. Here, a multiplex serology assay was used to detect CMV-specific IgG, IgM and IgA simultaneously. Antigen preparation and test methods were previously described elsewhere [21, 22]. Briefly, plasma samples diluted 1:1000 were tested for antibodies against 4 human CMV proteins (pp28, pp52, pp65 and pp150) bacterially expressed as glutathione *S*-transferase (GST) fusion proteins. The multiplex antibody detection approach was based on a GST capture immunosorbent assay in combination with fluorescent bead technology (Luminex Corporation, Austin, Texas) [22, 23]. The seropositivity threshold for each protein was set at a median fluorescence intensity (MFI) of 150 units and an individual was defined as CMV seropositive if two or more CMV-specific proteins were above the threshold. A validation of the CMV-specific multiplex assay was performed against Enzygnost anti-CMV/IgG and showed excellent sensitivity and specificity values (Brenner et al. in preparation).

### Data analysis

In order to assure that estimates derived from the GNHIES98 study are representative at the national level, survey weights were applied throughout the statistical analyses which accounted for the stratified and clustered sample design of the survey [20]. The survey weight takes into account the region, sex, and age distribution of the population of Germany in the year of the survey (1998). To ensure representativeness, the subpopulation with available data for CMV serostatus was compared to the total GNHIES population.

Analyses were conducted in a stratified dataset, in which men and women were analysed separately to account for gender differences. Univariate analysis was used to identify associations between sociodemographic factors and CMV seropositivity. Factors that were identified as possible influencing factors on CMV seropositivity in the literature and with a p-value <0.20 in the univariate analysis were included in the multivariable weighted binomial regression model. Interactions between factors were taken into consideration in the multivariable model. The final multivariable model included all factors that were associated with CMV seropositivity at a p<0.05 level in a forward stepwise selection approach. Results were expressed as weighted crude and adjusted prevalence ratios (PRs) with their 95% confidence intervals (95% CI). All analyses were done with STATA14.

## Results

### CMV seroprevalence in the adult population of Germany

The results are based on data from 6552 participants. Characteristics of the study population are shown in Table 1. The study population was representative of the adult population in Germany with an age range from 18 to 79 years and 51.5% of the participants being female. An analysis showed no significant differences regarding sociodemographic factors between the study population and the total GNHIES98 population (N = 7124).

**Table 1:** Study population characteristics, CMV seroprevalence study, GNHIES98, Germany (n = 6552 participants).

Variables		N	%
Total		6552	100
Age in years	17–29	1166	17.8
	30–39	1450	22.1
	40–49	1226	18.7
	50–59	1270	19.4
	60–69	940	14.4
	70–79	500	7.6
Sex	Male	3172	48.4
	Female	3380	51.6
Country of birth	German	5769	88.0
	Other	606	9.3
	Missing	177	2.7
Smoking status	Never smoking	2907	44.4
	Previous smoking	1395	21.3
	Current smoking	2097	21
	Missing	153	2.3
Region I in Germany	North	1679	25.6
	Middle	3008	45.9
	South	1865	28.5
Region II in Germany	East	2232	34.1
	West	4320	65.9
Education	Low	1121	17.1
	Middle	3668	56
	High	1587	24.2
	Missing	176	2.7
Attended child care	Yes	3224	49.2
	No	3140	47.9
	Missing	188	2.9
No of household members <18 yrs	0	4136	63.1
	> = 1	2233	34.1
	Missing	183	2.8
No of children grown up with	0	926	14.1
	> = 1	5442	83.1
	Missing	184	2.8
CMV serostatus	Negative	2867	43.3
	Positive	3685	56.7

**Fig 1:**
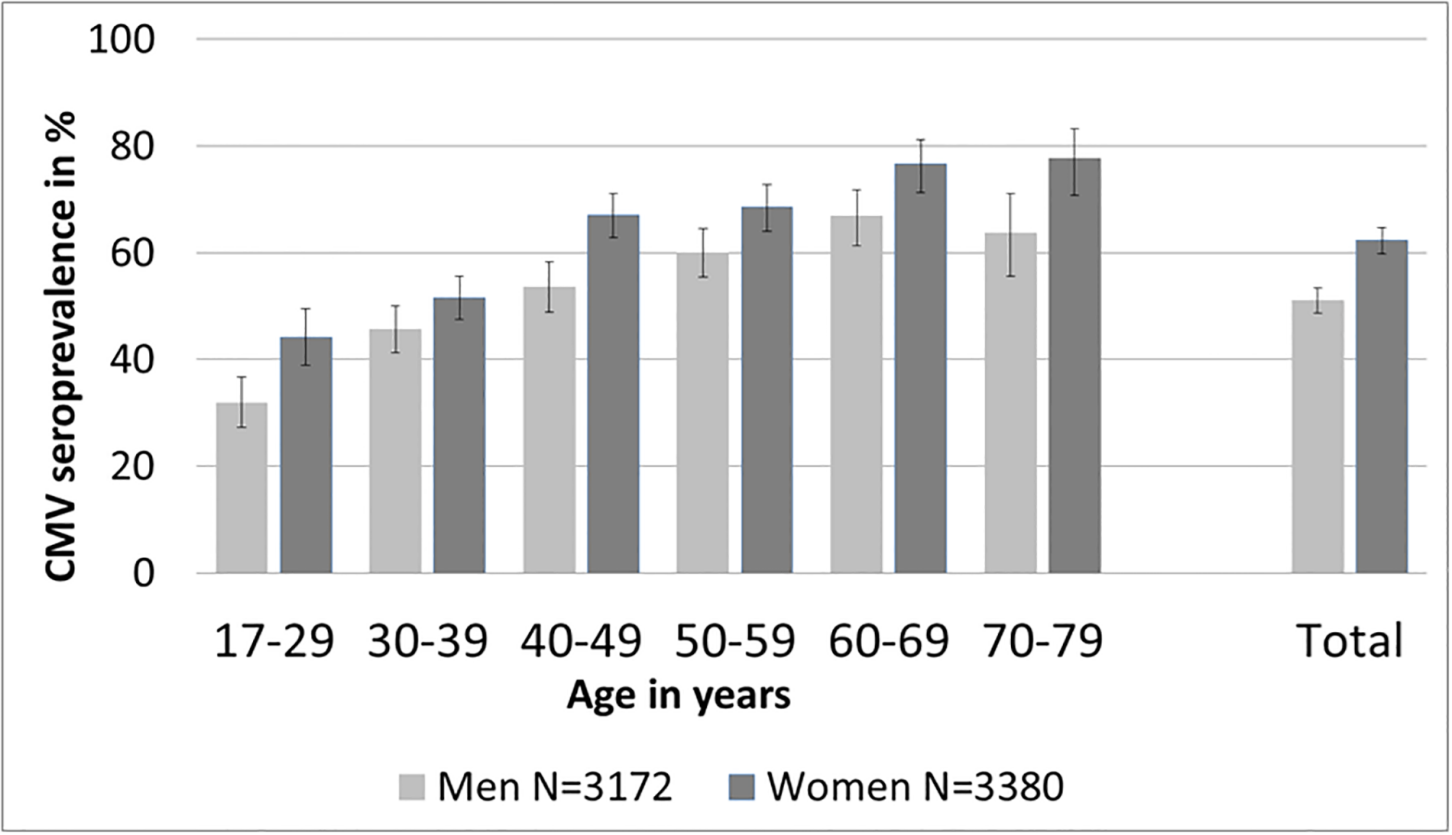
Estimated CMV seroprevalence (in percent) and 95% CI in adults in Germany, by age group and sex. In addition, overall seroprevalence and 95% CI by sex (men = light grey, women = dark grey) are shown on the right. Germany, n = 6552, sera collected 1998–1999.

Overall CMV seroprevalence in the adult population of Germany was estimated to be 56.7% (95% CI: 54.8–58.7%). In men, CMV seroprevalence was 51.0% (95% CI: 48.7–53.3%) and in women 62.3% (95% CI: 59.8–64.6%) Seroprevalence increased with age (Fig 1). In men, seroprevalence increased from 31.8% (95%CI: 27.3–36.8%) to 63.7% (95% CI: 55.6–71.1%) when comparing 18 to 29 with 70 to 79 years old individuals. In women, seroprevalence increased from 44.1% (95% CI: 38.8–49.5%) in 18 to 29 years old women to 77.6% (95% CI: 70.8–83.2%) in 70 to 79 years old women (Fig 1). In all age groups, CMV seroprevalence was higher in women than in men. Estimated CMV seroprevalence was higher in North Germany (Men: 52.4%, 95% CI: 48.4–56.5%; Women: 65.6% 95% CI: 61.4–69.6%) than in South Germany (Men: 47.1%, 95% CI: 43.3–51.0%; Women: 57.1% 95% CI: 52.8–61.3%). Total CMV seroprevalence in women of childbearing age (18–45 years) was 51.7% (95% CI: 47.8–54.3%). The study population included 34 women pregnant at the time of study participation. Of these, 13 (34%) were CMV seropositive.

### Factors associated with CMV seropositivity

The weighted crude and adjusted PR for men and women can be found in Table 2. In the multivariable model mutually adjusted for all other variables, the following factors were associated with CMV seropositivity in Germany in both, men and women: age (PR men: 1.02; women: 1.02), country of birth other than Germany (PR men: 1.76; women: 1.52;), current smoking (PR men: 1.11; women: 1.11), living in northern Germany (PR men: 1.15; women: 1.11), the number of household members under the age of 18 years (PR men: 1.09; women: 1.05;), and the level of education (PR men: 0.82; women: 0.90) (Table 2). Some factors were only associated with CMV seropositivity either in men or in women: attended child care during childhood (PR 0.91) was negatively associated with CMV seropositivity in men only, whereas in women, the number of siblings grown up with (PR 1.01) and living in East Germany (PR 1.14) were positively associated with CMV seropositivity. No significant terms of interaction between variables were identified. Residence in urban or rural regions and working with children (e.g. teacher, working in a kindergarten) was not associated with seropositivity in men or women, neither in uni- nor in multivariable analysis. Some variables that have been shown to be associated with CMV seropositivity in other studies, such as the number of sexual partners or income (and thereby socioeconomic status) were not included in this analysis because these variables were only available for less than 60% of the participants. The number of pregnant women (N = 34, age 20–41 years, median age 32 years) in this study was too small for further analysis.

**Table 2:** Results of univariable (crude PR) and multivariable model (adjusted PR) with CMV seropositivity as the dependent variable; data set stratified by gender; Germany (sera collected 1998–1999, n = 6552).

Variables		Men		Women	
		Crude PR (95%CI)	Fully adjusted PR (95%CI)*	Crude PR (95%CI)	Fully adjusted PR (95%CI)*
Age in years Country of Birth		1.01 (1.01–1.02)	1.02 (1.01–1.02)	1.01 (1.01–1.01)	1.02 (1.01–1.02)
	Germany	1 (Ref)	1 (Ref)	1 (Ref)	1 (Ref)
	Other	1.85 (1.70–2.02)	1.76 (1.62–1.92)	1.50 (1.41–1.60)	1.52 (1.41–1.63)
Smoking status	Non-smoking	1 (Ref)	1 (Ref)	1 (Ref)	1 (Ref)
	Previous smoking	1.27 (1.15–1.42)	1.10 (1.00–1.21)	0.92 (0.84–1.01)	1.02 (0.94–1.11)
	Smoking	1.11 (1.00–1.23)	1.11 (1.01–1.21)	0.95 (0.88–1.02)	1.12 (1.04–1.20)
Region I in Germany	South	1 (Ref)	1 (Ref)	1 (Ref)	1 (Ref)
	Middle	1.13 (1.02–1.25)	1.15 (1.05–1.27)	1.13 (1.03–1.23)	1.08(0.98–1.19)
	North	1.11 (1.00–1.24)	1.16 (1.04–1.29)	1.15 (1.04–1.27)	1.13 (1.03–1.24)
No of household members <18 yrs		1.03 (0.99–1.07)	1.07 (1.03–1.12)	0.96 (0.92–1.00)	1.05 (1.01–1.09)
Education	Low	1 (Ref)	1 (Ref)	1 (Ref)	1 (Ref)
	Middle	0.74 (0.67–0.81)	0.85 (0.77–0.93)	0.78 (0.73–0.83)	0.92 (0.86–0.98)
	High	0.75 (0.66–0.84)	0.82 (0.72–0.92)	0.77 (0.70–0.85)	0.90 (0.82–1.00)
Attended child care	No	1 (Ref)	1 (Ref)	1 (Ref)	ns^#^
	Yes	0.71 (0.65–0.78)	0.91 (0.83–1.00)	0.81 (0.75–0.87)	
Region II in Germany	West	1 (Ref)	ns^#^	1 (Ref)	1 (Ref)
	East	1.02 (0.93–1.11)		1.15 (1.04–1.19)	1.15 (1.08–1.23)
No of children grown up with		1.07 (1.05–1.09)	ns^#^	1.02 (1.01–1.03)	1.01 (1.00–1.02)

* Mutually adjusted for all other variables in the model,

^#^ ns = Variables were not significantly associated with CMV seroprevalence in the final model and therefore excluded

## Discussion

This is the first nationwide, representative CMV serosurvey in the adult population of Germany. Although these sera were collected in 1998, this population based CMV seroprevalence data are an important source for epidemiological modelling and they will serve as baseline data of longitudinal surveys in the future. In this study, 51% of men and 62% of women were positive for CMV-specific Ig; these data are comparable to seroprevalence data from France and the Netherlands [24, 25]. In contrast, populations in Portugal (77%), Sweden (83%) and Croatia (77%) seem to have slightly higher CMV seroprevalence [26–28].

In our study, age and country of birth were the most prominent independent factors associated with CMV seropositivity as it was shown in studies from other countries [1, 29, 30]. The increase of CMV seroprevalence with age is well known and results from cumulative exposure to CMV throughout life. The association between country of birth and CMV seroprevalence has been shown previously [1, 25, 31, 32]. Seroprevalence differences between countries may be explained by differences in the prevalence of key exposures related to CMV transmission: breastfeeding frequency and duration, crowding, childcare arrangements and sexual behaviours [1].

CMV seroprevalence is usually higher in women than in men, which indicates that the exposure to CMV might be partially different between genders. In most publications investigating factors associated with CMV seropositivity, gender is being adjusted for but not analysed separately. Using the stratified approach for gender the results shown here indicate that factors associated with CMV seropositivity indeed varied partially between men and women. The number of siblings was associated with CMV seropositivity only in women and not in men. One reason may be different playing behaviours and traditional role patterns, with women having been more involved in caring for their siblings in childhood and therefore more exposed to CMV shed by young children. In line with the high risk of CMV transmission from young children, the number of household members under the age of 18 years was associated with CMV seropositivity. Since the number of children raised was not available, the variable “number of household members under the age of 18 years” was used as a proxy. However, this proxy probably underestimated the real number of raised children. Young children constitute a well-known source of CMV because they often excrete large amounts of virus in their saliva and urine for a long time and therefore attending childcare is usually associated with higher CMV seroprevalence [30, 32, 33]. It is unclear, why in our study having attended childcare was not associated with CMV seropositivity in women and was associated with lower CMV seropositivity in men. In the past, child care settings differed in East and West Germany and also changed over time hindering interpretation. As in other studies, higher education was inversely associated with CMV seropositivity in this study [24, 25, 32, 34, 35]. Moreover it is known that smoking has an influence on the immune system and thereby on viral infections [36]. As in our study, smoking has also been shown to be associated with CMV seropositivity previously but it is still unclear if smoking has a direct effect on CMV infection or if it is a proxy for other lifestyle factors [37, 38]. Our results indicate that there were significant regional differences in the CMV seroprevalence in Germany as has been shown for other countries [25, 26, 39]. More lifestyle and behavioural data would be necessary to investigate what causes these regional differences.

In our study 51.7% of women of childbearing age were estimated to be CMV seropositive; thus, around half of women aged 18 to 45 years were susceptible for primary CMV infection. However, congenital CMV infections can occur both as a result of primary infection and after a reinfection or reactivation of latent CMV infection. A meta-analysis of Kenneson et al. estimated that 32% of primary infections and 1.4% of recurrent infections during pregnancy lead to congenital infection [40]. Due to high CMV seroprevalence globally, seropositive mothers account for the majority of CMV-induced permanent disabilities in children, even though the risk for congenital infection is higher in primary infections [40]. However, due to the relatively low seroprevalence, in Germany primary CMV infections during pregnancy are epidemiologically more important than reinfections and reactivation in CMV-seropositive pregnant women [41]. In a recent literature review Buxmann et al. estimated that annually 700–1400 children in Germany suffer from severe permanent disabilities due to congenital CMV [41]. Primary CMV infection (and probably reinfections) can be reduced significantly if women are educated about CMV transmission and preventive hygiene behaviours [10–14]. Despite the high disease burden, few women are aware of the risk of congenital CMV infection and CMV screening is not part of routine antenatal test [7–9, 42, 43]. If informed about preventive measures, women showed positive attitudes toward CMV prevention behaviours and perceived them as feasible [8]. These CMV prevention behaviours include washing hands after exposure to young children’s bodily fluids, not sharing food, cups, or other utensils with children, not putting a pacifier in the mouth after it had been in a child’s mouth, and not kissing children on the lips [8, 14]. The results from this study suggest that many women of child-bearing age are at risk of primary CMV infection and that there are no easily identifiable high-risk groups.

To date, there is no vaccine available that prevents primary or reactivation of CMV infection even though a vaccine would be the best chance to reduce the burden of CMV infection. However, several vaccine candidates are in clinical development, and a vaccine against CMV was classified as a top priority by “The National Vaccine Advisory Committee” in the US in 2004 which has triggered commercial interest [15–19]. In order to develop public health recommendations regarding the use of a CMV vaccine once available in the future, representative epidemiological data on the susceptibility of the population and the burden of CMV infection are essential. Since there are no population-based seroprevalence data for CMV antibodies available for Germany, the present study was conducted. Knowledge about population-based age-stratified CMV prevalence is necessary for the design of age-specific vaccination strategies [44]. Therefore population based surveys, such as the GNHIES98, provide useful seroprevalence data to be included in transmission models and to inform future vaccination strategies. Beyond the primary goal of reducing congenital CMV infection, the reduction in CMV transmission achieved by CMV vaccination could have further indirect benefits in terms of lowering CMV incidence in the immunocompromised and the elderly in an ageing population [44]. Meanwhile, CMV screening during pregnancy and educating women about CMV risk reduction measures could reduce congenital CMV infections with its serious consequences.

CMV seropositivity in our study was measured by GST-based multiplex serology assay which showed excellent sensitivity and specificity values in a validation study but may not be identical to estimates measured using other assays. The use of an assay which detects IgG, IgA and IgM simultaneously achieves high sensitivity but unfortunately it is not possible to analyse the different Ig-classes separately.

A major strength of this study was the use of a representative population-based sample to determine CMV seroprevalence. Because of the large sample size and the population-wide weighted sampling procedure the study also had sufficient statistical power to enable a reliable multivariable analysis of factors associated with CMV seropositivity. Even though a weighting factor was used in the analysis and a high response of 61% among eligible persons was achieved, some bias might be present since institutionalized persons and persons with inadequate German language skills were excluded. For this reason, the subpopulation of migrants in this study is not representative for migrants in Germany. In addition, for some factors, the individual status at the time of the survey may not reflect past exposure.

One important limitation is the age of the data used for this study. The sera were collected in 1998 and the CMV seroprevalence in the present population in Germany might have changed since then. Even though it can be assumed that CMV seroprevalence did not change substantially in the last 20 years (due to the long co-evolution between humans and human CMV [45]), further seroprevalence studies are necessary to confirm CMV seroprevalence in the present population in Germany. Due to changing circumstances (German reunification), lifestyle and behaviours (e.g. higher level of mobility of people moving around Germany, higher rate of children visiting day-care facilities) might have changed substantially in the population of Germany over the last 20 years which might affect CMV seroprevalence as well. Especially the immigration of refugees in the recent years should be considered in future surveys. Studies in the US showed no changes in the CMV seroprevalence of the total population, whereas studies in pregnant women only showed decreasing seroprevalence in Japan and increasing CMV seroprevalence in Norway [39, 46–48]. Since the participants in GNHIES98 constitute the baseline cohort for health interviews and examinations that were conducted in 2008–2011, the results from this study could also provide an excellent baseline for a longitudinal serosurvey. Longitudinal analysis would be essential to investigate if CMV seroprevalence changed over the last 20 years and if so which factors were associated with it.

## Conclusion

In conclusion, our study constitutes the first population-based seroprevalence data based on a large sample representative for the adult population living in Germany. These data indicate that a substantial proportion of women in childbearing age were susceptible to primary CMV infection. Further seroprevalence studies with more recent data are necessary to evaluate CMV seroprevalence in the German population and to better understand the epidemiology of CMV infection. As long as no effective vaccine is commercially available, the primary prevention measure should be educating women about CMV risk reduction measures.

## Abbreviations

CI: Confidence Interval
CMV: Cytomegalovirus
DEGS: German Health Interview and Examination Survey for Adults
DKFZ: German Cancer Research Center
GNHIES98: German National Health Interview and Examination Survey 1998
Ig: Immunoglobulin
No: Number
pp150: Phosphoprotein 150
pp28: Phosphoprotein 28
pp52: Phosphoprotein 52
pp65: Phosphoprotein 65
PR: Prevalence ratio
Ref: Reference
RKI: Robert Koch-Institute
